# Demethylzeylasteral (T-96) Treatment Ameliorates Mice Lupus Nephritis Accompanied by Inhibiting Activation of NF-κB Pathway

**DOI:** 10.1371/journal.pone.0133724

**Published:** 2015-07-24

**Authors:** Qiongyi Hu, Chunxin Yang, Qiang Wang, Haiying Zeng, Wanzhang Qin

**Affiliations:** 1 Department of Dermatology, Zhongshan Hospital, Fudan University, 180 Fenglin Road, Shanghai, 200032, P.R. China; 2 Department of Pharmaceutical Chemistry, Zhongshan Hospital, Fudan University, 180 Fenglin Road, Shanghai, 200032, P.R. China; 3 Department of Pathology, Zhongshan Hospital, Fudan University, 180 Fenglin Road, Shanghai, 200032, P.R. China; Penn State University, UNITED STATES

## Abstract

**Background:**

Inflammation plays a vital role in the pathogenesis in lupus nephritis (LN), which is largely attributable to the activation of nuclear factor kappa B (NF-κB) signal pathway. NF-κB up-regulates pro-inflammatory mediators, such as TNF-α, cyclo-oxygenase-2 (COX-2) and ICAM-1, and promotes macrophage infiltration into renal tissue, further inducing the progression of LN. Over the past 30 years, research has demonstrated that *Tripterygium wilfordii* Hook F (TWHF) possesses potent anti-inflammatory and immunosuppressive activities, and that demethylzeylasteral (T-96), an extract of TWHF, may be one of the responsible compounds. Here, we investigate the pharmacodynamic role and therapeutic mechanism by which T-96 suppresses inflammation and reduces renal pathology in the lupus-prone MRL/lpr mice.

**Methods:**

Forty-eight MRL/lpr mice were equally randomly divided into 6 groups (1.2, 0.6 or 0.3 mg/10g T-96, 0.022 pills/10g kang lang chuang san (one of Traditional Chinese herb as positive control), 0.125 mg/10g prednisone and 0.1 ml/10g normal saline as the LN disease control group). Also, eight WT C57BL/6 mice were used as normal control. After treatment by gavage with 0.10 ml/10g/day volumes for 8 weeks, all mice were sacrificed and renal tissues were collected. The amount of 24 h proteinuria and the levels of anti-dsDNA antibody in serum were assessed respectively at weeks 0, 4 and 8. Inflammation, cytokines and NF-κB levels were assessed by histological examinations, immunohistochemical analyses and Western blot analyses.

**Results:**

In comparison with untreated MRL/lpr mice, mice treated with 1.2 and 0.6 mg/10g of T-96 showed a significant improvement in 24 h proteinuria and the levels of anti-dsDNA antibody in serum. In addition, T-96 reduced the secretion of pro-inflammatory mediators such as TNF-α, COX-2 and ICAM-1, and the infiltration of macrophages in renal tissue. Moreover, T-96 significantly suppressed phosphorylations of cytoplasmic IKK and nuclear p65.

**Conclusion:**

This study suggests that T-96 exhibits reno-protective effects in LN accompanied by inhibiting the activation of NF-κB, reducing the downstream pro-inflammatory mediators and thus restricting macrophage infiltration. Because of these potent properties, T-96 should be considered as a promising therapeutic drug for LN.

## Introduction

Systemic lupus erythematosus (SLE) is a chronic autoimmune disease that involves multiple organs with a variety of manifestations such as rash, nephritis and arthritis. These symptoms are manifested primarily in females between the ages of 15 and 50 [1]. Lupus nephritis (LN), one of the most common and severe complications in SLE, is characterized by glomerulonephritis and tubulointerstitial inflammation with the immune-complexes depositing in the renal tissue [2]. The involvement of LN, especially the type of proliferative glomerulonephritis significantly reduced the survival and life expectancy of LN patients [3]. Therefore, there is an urgent need to find an effective treatment aiming at new targets for SLE patients.

Inflammation plays a vital role in the pathogenesis in LN, with the macrophages playing a primary role [4,5]. Studies have identified macrophages, located throughout the interstitium and in and around glomeruli, as the source of critical markers that predict proteinuria onset, progression, remission, and impending relapse in LN [6,7]. In recent years, there have been considerable advances in the treatment of LN. Drugs targeting renal macrophages may have the potential to become a treatment option with significantly improved efficacy and safety profiles [8,9]. Nuclear factor kappa B (NF-κB), arguably the best-studied inducible transcription factor over the past 25 years, is widely accepted as a critical regulatory modulator of various biological processes including innate and adaptive immunity and also inflammation [10,11]. The dysregulation of NF-κB activation is considered to drive many human diseases, especially those involving inflammatory and immune responses, and recent studies suggest that NF-κB may play a prominent role in the onset and progression of LN as well [12–15]. In its inactive state, NF-κB usually exists in the cytoplasm bound to its inhibitory protein, inhibitor of κB (IκB), which functions to mask the nuclear localization sequence of NF-κB. In response to a diversity of stimuli, IκB is phosphorylated by the activation of IκB kinase (IKK), subsequently ubiquitinated and degraded, thus leading to the release of NF-κB. As a result, activated NF-κB dimers translocate to the nucleus, bind to the specific DNA sequences, and induce target proteins to mediate inflammatory and immune responses [16].

*Tripterygium wilfordii* Hook F (TWHF), commonly known as “lei gong teng” or “thunder god vine”, is widely distributed in China, Korea, and Japan [17]. Since its debut in the 1960s in China, it has been widely used as a therapeutic for autoimmune and inflammatory diseases including rheumatoid arthritis, ankylosing spondylitis, SLE and psoriasis [18–21]. The pharmacological mechanisms by which specific extracts of TWHF function remain unclear, while the major therapeutic effects of TWHF have been attributed to diterpenoids and triterpenoids, such as triptolide, tripterine, etc [22]. Demethylzeylasteral (T-96) is a triterpenoid that has recently been isolated from the root xylem of TWHF [23]. Over the past 30 years, numerous studies have indicated that TWHF exhibits potent anti-inflammatory and immunosuppressive activities [17], through restraining the functions of pro-inflammatory cells such as macrophages, dendritic cells, T and B lymphocytes, and by decreasing the production of some of their pro-inflammatory mediators such TNF-α, IL-6, IL-8, IL-1 and IL-12 [24]. In addition, TWHF extracts possess an immunomodulatory effect via the activation of the “IKK-IκB-NF-κB” signal pathway to initiate anti-inflammatory effects [25].

Although T-96 has been reported to exert immunosuppressive effects in a rat kidney transplantation model [23], the anti-inflammatory effects and pharmacological mechanisms of T-96 remain unclear. In the present study, we hypothesize that T-96 can prevent the development of LN in the lupus-prone MRL/lpr mice and demonstrate that it inhibits activation of renal NF-κB signaling and reduces macrophage infiltration.

## Materials and Methods

### Drugs

**Fig 1:**
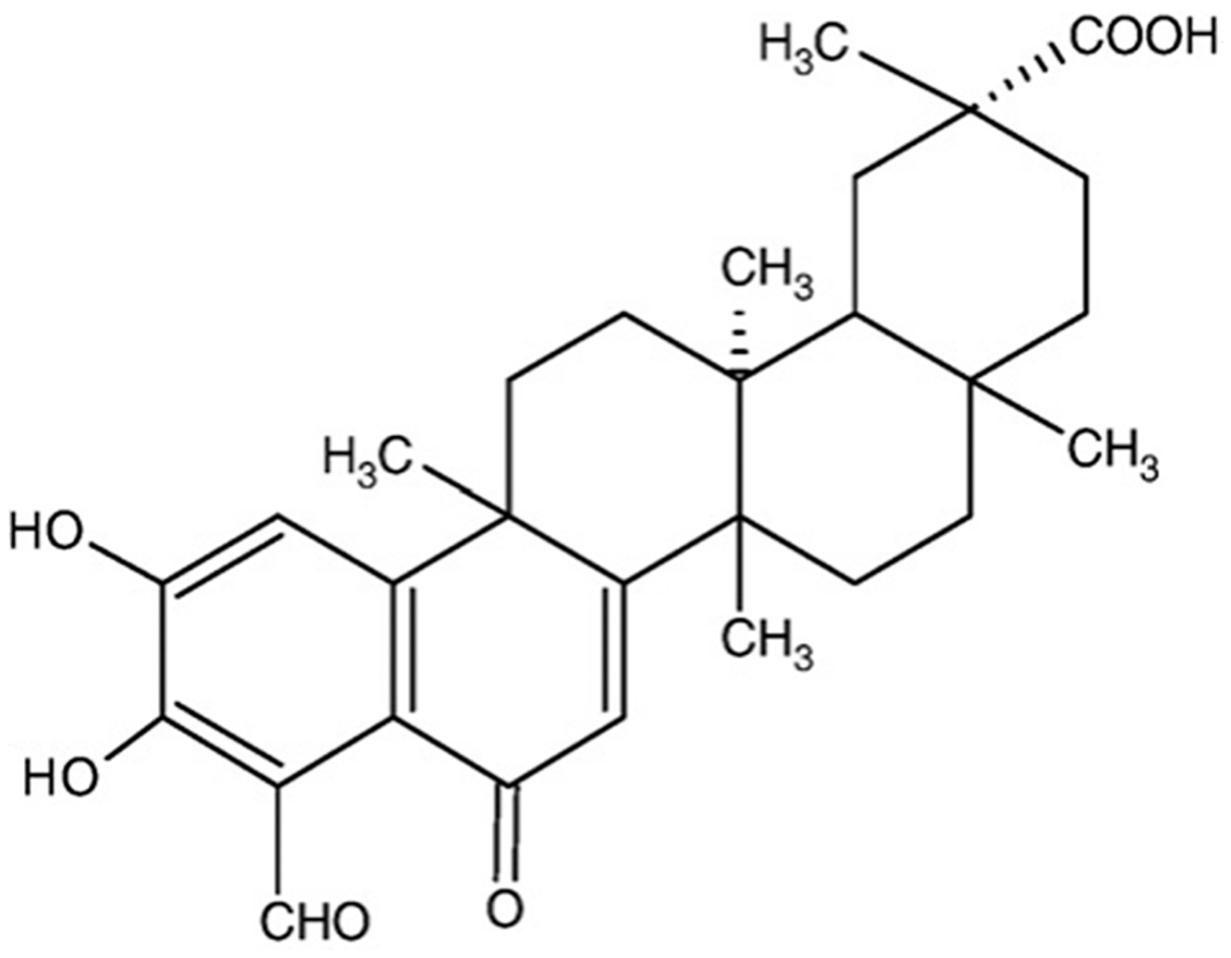
The chemical structure of demethylzeylasteral.

Demethylzeylasteral (T-96) (yellow amorphous powder, Molecular formula: C_29_H_36_O_6_, MW: 480) was isolated and provided by Professor C.X. Yang (Department of Pharmaceutical Chemistry, Zhongshan Hospital, Fudan University, Shanghai, China). T-96 was isolated from the crushed root bark of TWHF by means of ethyl acetate extraction and silica gel column chromatography utilizing a chloroform-methanol gradient elution technique. Subsequent concentration and recrystallization yielded yellow T-96 crystals. The chemical structure of demethylzeylasteral is shown in Fig 1. Kang lang chuang san (one of Traditional Chinese herb) was purchased from Huaying Pharmaceutical Co., Ltd (Hebei, China, No. Z1990006). Prednisone was purchased from SINE Pharmaceutical Co., Ltd (Shanghai, China, No. 101101), 0.9% normal saline was purchased from Shanghai Changzheng Fumin Jinshan Pharmaceutical Co., Ltd (Shanghai, China, No. 11080907).

### Animals

Forty-eight female MRL/lpr mice (body weight: 38.5 ± 2.7g and age: 20 weeks old) and eight WT C57BL/6 mice (body weight: 21.4 ± 1.0g and age: 8 weeks old) were purchased from the Shanghai SLAC Laboratory Animal Center under the license from the home office in accordance with the Animal (Scientific Procedures) Act 1986 (Shanghai, SCXX 2007–006, China). Mice were maintained under specific pathogen-free conditions in the Animal Center, where the mice were kept with a 12-h light/dark cycle and with access to standard water and food ad libitum. 48 MRL/lpr mice were randomly equally divided into 6 groups, including Group A to F (group A = 1.2 mg/10g T-96, group B = 0.6 mg/10g T-96, group C = 0.3 mg/10g T-96, group D = 0.022 pills/10g kang lang chuang san, group E = 0.125 mg/10g prednisone, group F = 0.1 ml/10g normal saline as model group). Preparation of the solutions administered to animals by gavage: dissolve T-96 in anhydrous ethanol, q.s. with water to 12 mg/ml, 6 mg/ml and 3 mg/ml suspensions. The clinical equivalent dose used in mice can be converted according to the conversion co-efficients table for the dose per kilogram of animal and human body weight [26]. T-96 doses used (0.3~1.2 mg/10g/day) were based on the results of our previous study (Q. Wang, C.X. Yang: unpublished observations). Also, eight WT C57BL/6 mice were used as normal control (Group N). All groups were gavaged 0.1 ml/10g/day for 8 weeks. Also, body weight, the size of lymph node and the condition of skin fur were detected at week 0, 4, 8. After treatment for 8 weeks, all mice were sacrificed under diazepam anesthesia. At week 8, the kidney samples were collected, fixed in 4% neutral-buffered formalin and embedded in paraffin. Additional kidney samples were frozen in liquid nitrogen and stored at -80°C. All experimental protocols described in this study were approved by the Animal Ethical Committee of Zhongshan Hospital, Fudan University.

### Reagents

Rabbit monoclonal antibodies against mouse p-p65 and p-IKK antibody were purchased from Cell Signaling Technology (USA), rabbit polyclonal antibodies against mouse CD68, IL23, TNF-α, COX-2 and ICMA-1 were purchased from Abcam (Cambridge, UK), mouse monoclonal antibodies against tubulin were purchased from Beyotime Institute of Biotechnology (Shanghai, China) and rabbit monoclonal antibodies against lamin B1 were purchased from Proteintech (Wuhan, China). HRP-conjugated secondary antibody was purchased from Cell Signaling Technology (USA). 3,3-diaminobenzidine (DAB) kit was purchased from Maixin Biological Company (Fuzhou, China).

### Detection of the urinary protein

Urine samples at 24 h were collected in metabolic cages every four weeks during the period of experiment before sacrifice, and centrifuged at 2000 xg for 5 min to remove any particulates. The supernatant was collected and frozen at -20°C until measurement. 24 hour urinary protein was detected by Coomassie brilliant blue test.

### Evaluation of serum anti-dsDNA antibody levels by ELISA

Blood samples were drawn from the ophthalmic venous plexus every four weeks and the levels of anti-dsDNA antibodies in serum was determined by enzyme-linked immunosorbent assay (ELISA) as previously described [27] according to the manufacturer’s protocol.

### Histopathological examinations (H&E and PAS stainings)

For microscopic examination, 3 μm-thick formalin-fixed and paraffin-embedded sections of kidney tissues were stained with hematoxylin and eosin (H&E) and periodic acid-Schiff (PAS) stains. The scores of pathological activity index (AI) for LN was semi-quantitatively graded on a scale of 0–18 as reported previously [28]. In a brief, histological abnormalities, including the glomerular (cresents, mesangial region, capillary loops), tubular, interstitial and vascular damage were scored separately for each kidney using a semi-quantitative scale from 0–3, where 0 = absent, 1 = mild, 2 = moderate, 3 = severe.

### Immunohistochemical analyses

As described in detail previously [29], 3 μm-thick sections were made and initially deparaffinized by xylene and dehydrated with ethanol. Endogenous peroxidase activity was blocked by 3% hydrogen peroxide in methanol at room temperature for 15 min, and then slides were dipped into ethylenediamine tetraacetic acid to restore antigens. After cooling to room temperature, sections were incubated with the diluted primary antibodies (p-p65 antibody, p-IKK antibody, CD68 antibody, IL23 antibody, TNF-α antibody, COX-2 antibody, ICAM-1 antibody) (1:100) in a wet box at 4°C overnight. The next day, sections were incubated with secondary antibody and EnVision (ChemMafe EnVision^+^/HRP). The reaction was then visualized with DAB. Sections were counterstained with hematoxylin, dehydrated, and evaluated under light microscopy (BX51 Olympus, Japan). Renal tissue cells containing yellow granulation in the endochylema or nucleus were considered as positive. The number of positive cells was counted with Q500IW image analysis system (BX51 Olympus, Japan) and Image-Pro Plus 6.0 software (Media Cybernetics Inc., Bethesda, MD, USA).

### Western blot analyses

Nuclear and cytoplasmic proteins were obtained with a commercial nuclear extraction kit (KeyGEN Biotech, China) according to the manufacturer's instructions. Cell lysate protein concentration was detected using the BCA Protein Assay kit (Beyotime Institute of Biotechnology, Shanghai, China). Equal quantities of protein (40 μg) were separated by SDS-PAGE and transferred to PVDF membrane (Millipore, USA). After blocking with 5% non-fat milk in TBST (TBS with 0.1% Tween-20, pH 7.4) at room temperature for 2 h, membranes were incubated with primary antibodies: anti-p-p65 (1:1000), anti-p-IKK (1:1000) at 4°C overnight. Membranes were washed three times and incubated with HRP-conjugated secondary antibody at room temperature for 2 h. Anti-tubulin antibody and anti-lamin B1 antibody were used as internal controls. Peroxidase was visualized using an enhanced chemiluminescence system (ECL) (Thermo, USA). Bands were quantitated using Image J 1.48 software (Bio-Rad, USA), and results are expressed as fold change relative to the internal control.

### Statistical analysis

All data are expressed as mean ± SD (standard deviation). The significance of results obtained from the control and treated groups was performed using ANOVA (one-way analysis of variance) or the nonparametric Wilcoxon rank-sum test by SPSS 20.0 software. *P* value less than 0.05 was considered statistical difference.

## Results

### The observations of body weight, the size of lymph node and the condition of skin fur

Body weight, the size of lymph node and the condition of skin fur were recorded at week 0, 4, 8. There were no significantly differences in body weights, the size of lymph node and condition of skin fur among the groups of mice at week 0, 4, 8 (data not shown).

### T-96 treatment reduced 24 h proteinuria

**Fig 2:**
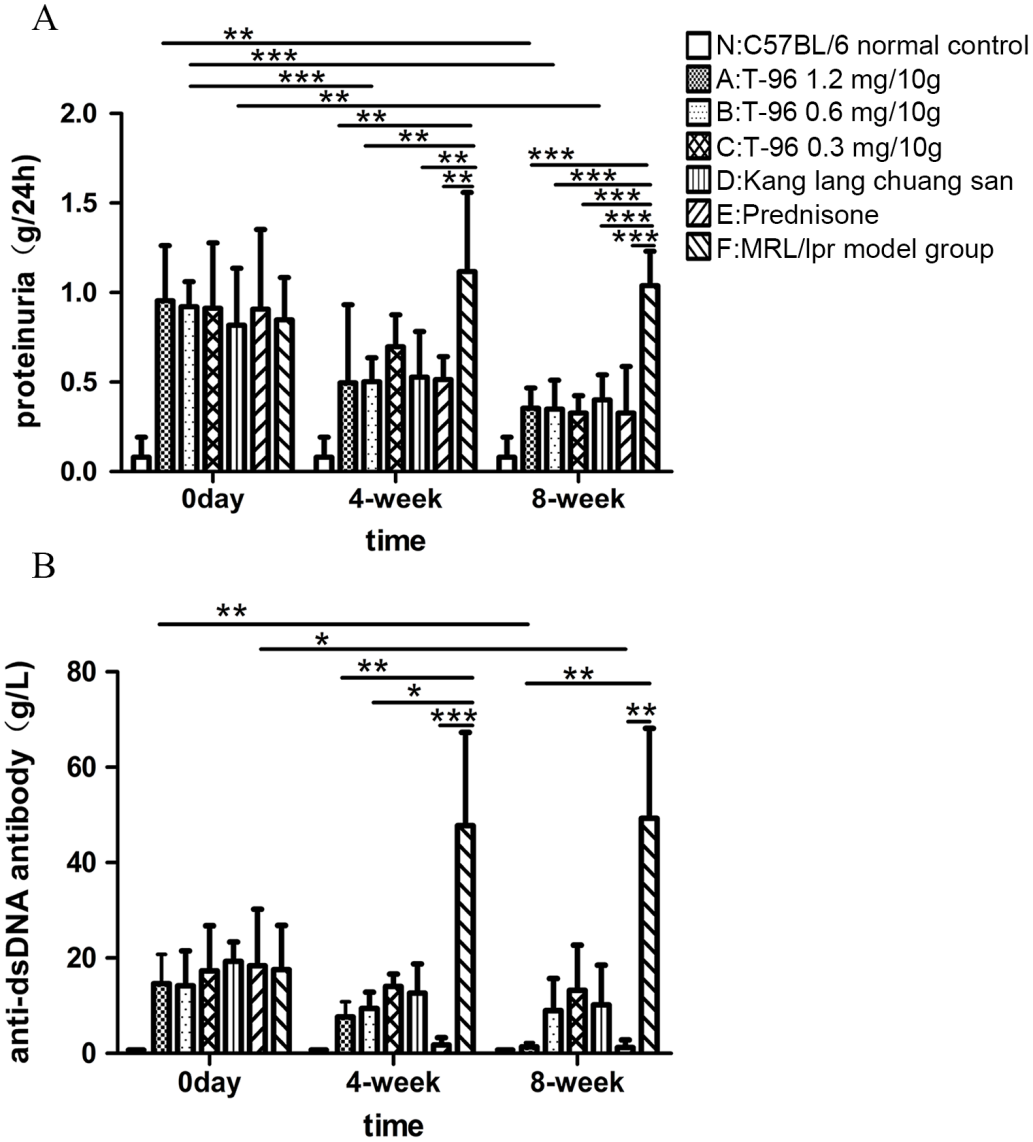
T-96 improves 24 h proteinuria and anti-dsDNA antibody in serum of MRL/lpr mice. (A) 24 hour urinary protein was detected by Coomassie Brilliant Blue test at weeks 0, 4 and 8. (B) Anti-dsDNA antibody levels in serum were measured by ELISA at weeks 0, 4 and 8. Data were expressed as mean ± SD. * indicates P < 0.05, ** indicates P < 0.01, *** indicates P < 0.001.

To investigate whether T-96 had an effect on the development of renal disease over time, proteinuria was determined every 4 weeks during treatment with T-96 from week 0 to week 8. Group F, the normal saline-treated MRL/lpr mice, showed a progressive rise of 24 h proteinuria over time compared with group N, C57BL/6 normal control (Fig 2A). However, at week 4, the amount of 24 h proteinuria was significantly decreased in mice receiving 1.2 and 0.6 mg/10g T-96 relative to the normal saline-treated MRL/lpr mice (Fig 2A; both p < 0.01). At week 8, mice treated with 1.2 to 0.3 mg/10g T-96 had markedly less 24 h proteinuria than the normal saline-treated MRL/lpr mice (Fig 2A; all p < 0.001). Additionally, the amount of 24 h proteinuria in mice treated with 1.2 mg/10g T-96 fell from 0.96 ± 0.31 g/24h at week 0 to 0.34 ± 0.11 g/24h at week 8 (Fig 2A; p < 0.01); at week 0, the amount of 24 h proteinuria was 0.92 ± 0.14 g/24h in mice with 0.6 mg/10g T-96, the number declined dramatically to 0.50 ± 0.13 g/24h by week 4 and continued to declined to 0.35 ± 0.16 g/24h by week 8 (Fig 2A; p < 0.001). Mice treated with kang lang chuang san had lower proteinuria than the normal saline-treated MRL/lpr mice at week 8; and the amount descended significantly from week 0 to week 8 (Fig 2A). Furthermore, there was a significant difference in the 24 h proteinuria between T-96 and prednisone treatment at week 4, as well as week 8 (Fig 2A). Taken together, T-96 demonstrated a significant proteinuria reduction both in a time and concentration-dependent manner.

### T-96 treatment reduced the levels of anti-dsDNA antibody in the serum

The levels of anti–dsDNA antibody in serum are closely related to disease activity in SLE and serve as the primary criterion by which treatment efficacy is measured. In C57BL/6 normal controls (Group N), showed an absence of anti-dsDNA antibody in serum over time, indicating an absence of LN, whereas the levels of anti-dsDNA antibody in normal saline-treated MRL/lpr mice displayed an obvious evidence of LN, increasing from 17.56 ± 9.25 g/L at 0 week to 47.80 ± 19.52 g/L at week 4, further to 49.27 ± 18.89 g/L at week 8 (Fig 2B). In contrast, administration of 1.2 and 0.6 mg/10g T-96 significantly dropped anti-dsDNA antibody levels compared to normal saline-treated MRL/lpr mice after treatments for 4 weeks (Fig 2B; 1.2 mg/10g T-96 p < 0.01, 0.6 mg/10g T-96 p < 0.05, prednisone p < 0.001). In addition, the levels of anti–dsDNA antibody in mice with 1.2 mg/10g T-96 were also lower than the normal saline-treated MRL/lpr mice at week 8 (Fig 2B; p < 0.01). Prednisone had its expected effect, reducing the levels of anti-dsDNA antibody from 18.40 ± 11.82 g/L at 0 week to 1.23 ± 1.63 g/L at week 8 (Fig 2B; p < 0.05); and the levels of anti-dsDNA antibody were remarkably lower than that in MRL/lpr model group at week 4 and week 8, respectively (Fig 2B; p < 0.001 at week 4 and p < 0.01 at week 8). In conclusion, T-96 demonstrated that it could attenuate nephritis in systemic lupus erythematosus.

### T-96 treatment attenuated renal inflammatory responses (renal histological lesions, macrophage infiltration, IL23 expression and secretion of pro-inflammatory mediators)

**Fig 3:**
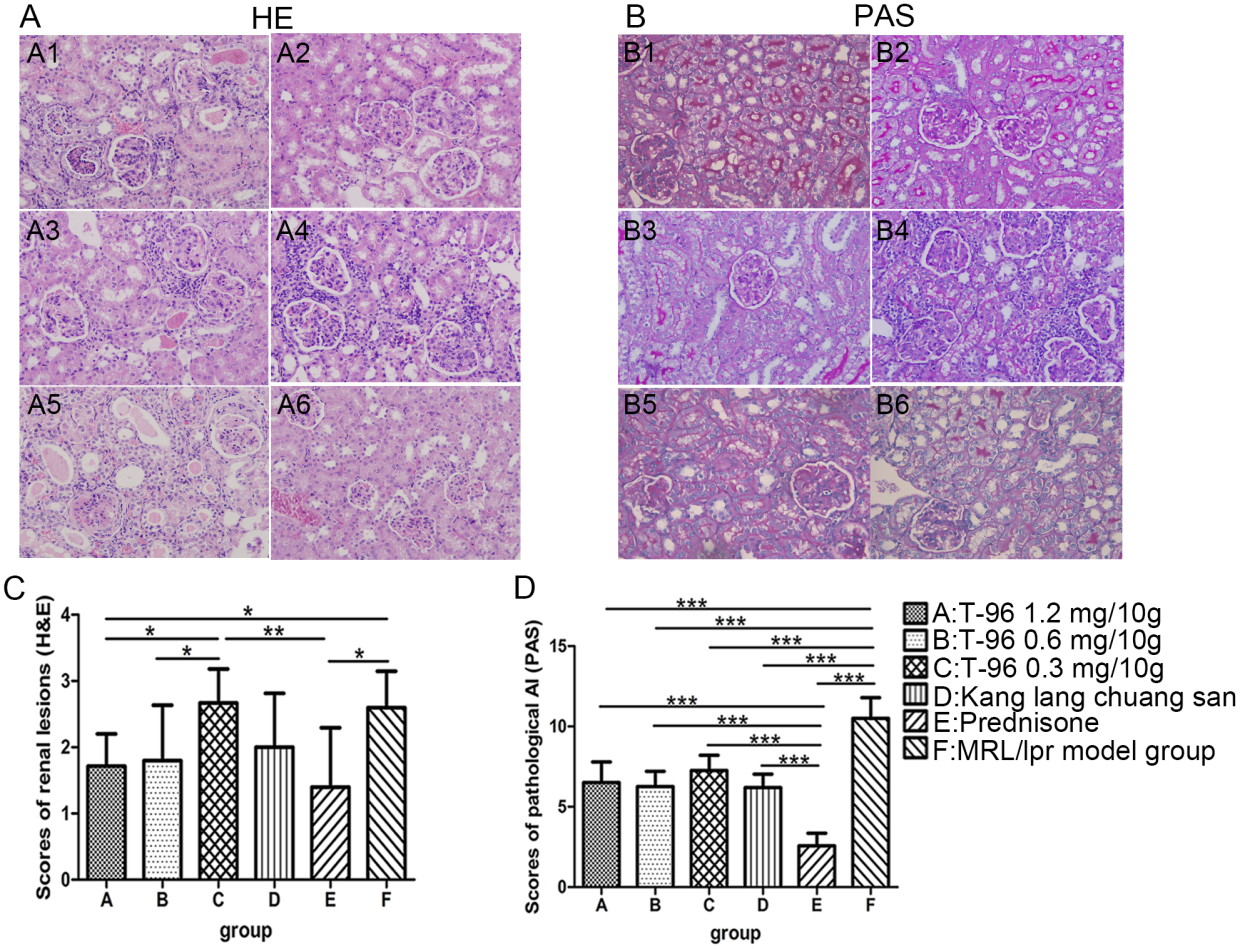
T-96 attenuates renal lesions in MRL/lpr mice. (A-B) Kidneys were collected at week 8, and stained with hematoxylin-eosin stain (H&E) (A) and Periodic Acid-Schiff stain (PAS) (B) (10 x 20). (C-D) The scores of renal lesions in H&E sections (C) and the scores of pathological activity index (AI) in PAS sections (D) were semi-quantitatively measured. Data were expressed as mean ± SD. * indicates P < 0.05, ** indicates P < 0.01, *** indicates P < 0.001.

**Fig 4:**
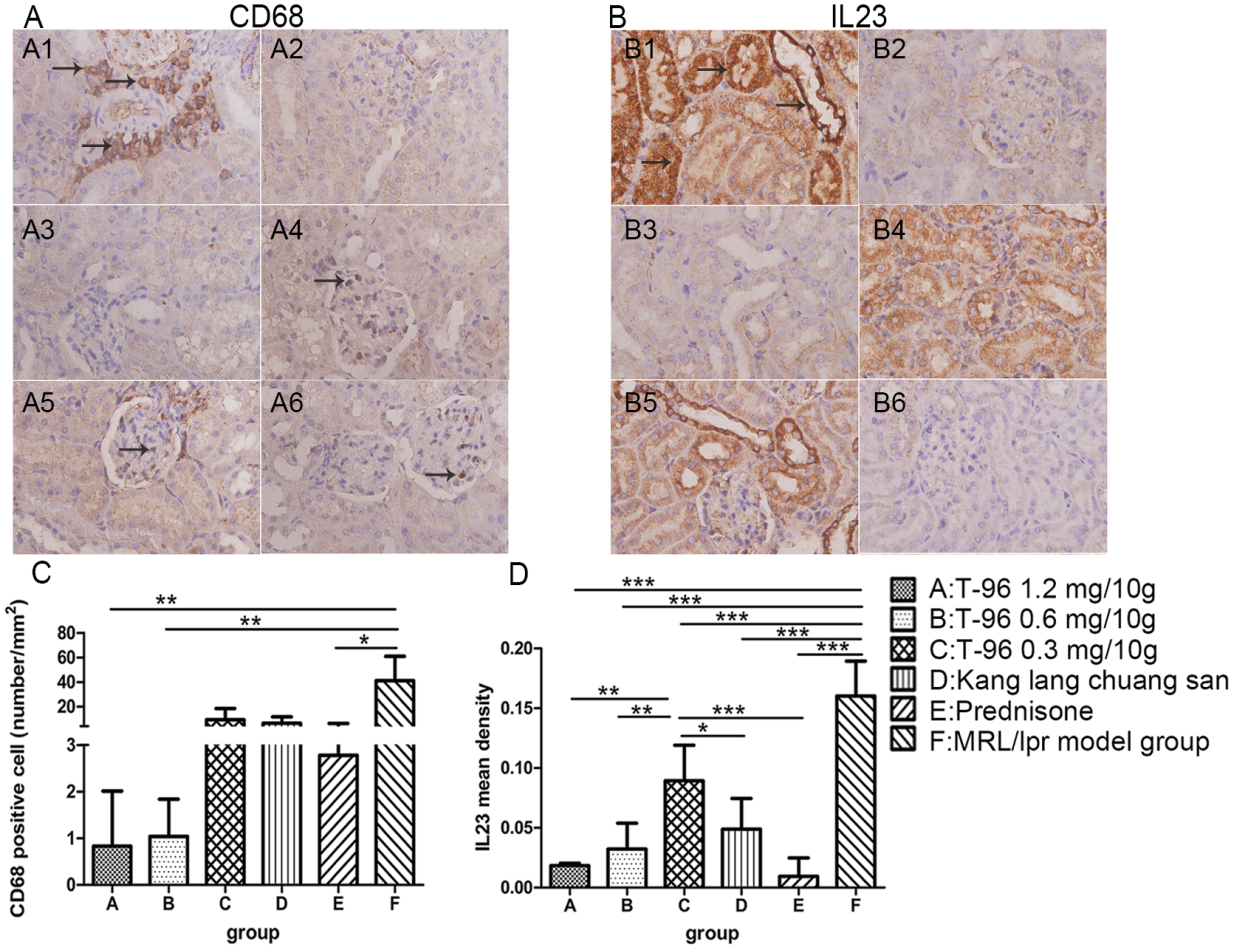
T-96 treatment inhibits infiltration of CD68^+^ macrophages and IL23 expression. CD68^+^ macrophages (A) and IL23 expression (B) in mice renal tissue after 8 weeks treatment were assessed by immunohistochemistry staining (10 x 40). CD68 and IL23 were indicated respectively by arrows. (C) The number of CD68^+^ macrophages per mm^2^ was measured. (D) Mean density of IL23 was measured by Image-Pro Plus v 6.0. Data were expressed as mean ± SD. * indicates P < 0.05, ** indicates P < 0.01, *** indicates P < 0.001.

**Fig 5:**
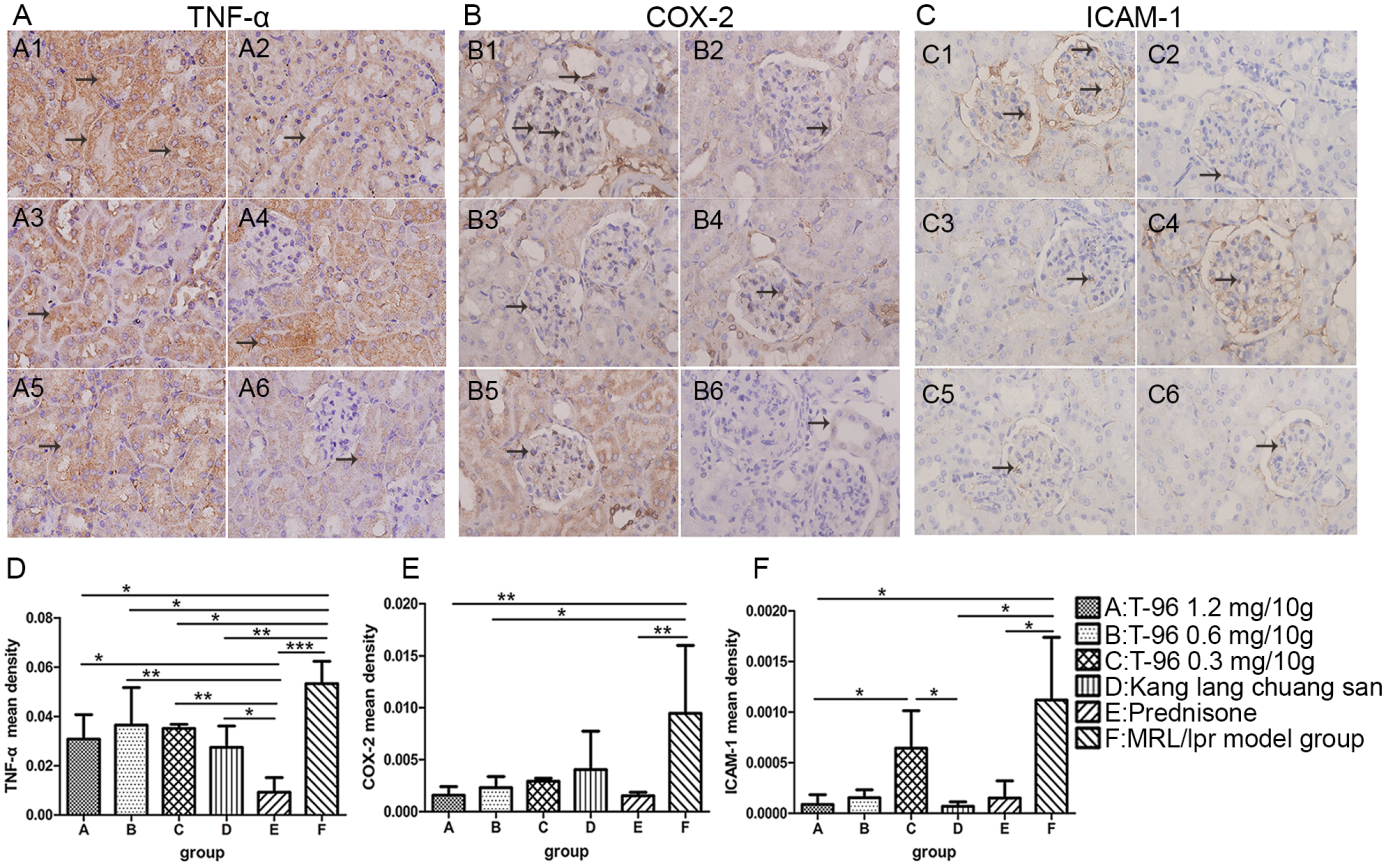
T-96 treatment inhibits pro-inflammatory mediators’ release of TNF-α, COX-2 and ICAM-1. The productions of TNF-α (A), COX-2 (B) and ICAM-1 (C) in renal tissue after 8 weeks treatment were identified by immunohistochemistry staining (10 x 40). TNF-α, COX-2 and ICAM-1 were indicated respectively by arrows. (D-F) Mean density of TNF-α (D), COX-2 (E) and ICAM-1 (F) were measured by Image-Pro Plus v 6.0. Data were expressed as mean ± SD. * indicates P < 0.05, ** indicates P < 0.01, *** indicates P < 0.001.

To assess inflammation in MRL/lpr mice, we analyzed pathological changes in the renal tissues. H&E and PAS stainings revealed histological abnormalities in glomeruli, tubule-interstitium and in vessels. Glomerulonephritis, including mesangial cell proliferation, mesangium expansion, crescent formation, and necrosis of capillary loops as well as tubule-interstitial lesions and vasculitis were readily detected in normal saline-treated MRL/lpr mice, manifesting a typical model of LN (Fig 3A1, 3B1, 3C and 3D; 2.60 ± 0.55 of renal lesions and 10.50 ± 1.29 of pathological AI). In contrast, T-96 treatment with 1.2 mg/10g notably ameliorated the lesions of renal tissue, with less damage to glomeruli, tubule-interstitium and vessels relative to normal saline-treated MRL/lpr mice (Fig 3A2, 3B2, 3C and 3D; scores of renal lesions p < 0.05, scores of pathological AI p < 0.001). Mice treated with 0.6 mg/10g T-96 tended to have mild glomerulonephritis, cast formation and inflammation in interstitium, with lower scores of renal lesions and pathological AI than the normal saline-treated MRL/lpr mice, though there were no significant differences in the scores of renal lesions (Fig 3A3, 3B3, 3C and 3D; AI p < 0.001). However, 0.3 mg/10g T-96 and Kang lang chuang san treatment didn’t significantly remit renal damage compared with the normal saline-treated MRL/lpr mice (Fig 3A4, 3A5, 3B4, 3B5, 3C and 3D; all p > 0.05). As expected, prednisone treatment markedly attenuated renal pathology, with mild glomerular mesangial proliferation, cast and interstitium infiltration (Fig 3A6, 3B6, 3C and 3D; both p < 0.001). The infiltration of inflammatory cells was further demonstrated by immunohistological staining for CD68^+^ cells. There was a predominant population of CD68^+^ macrophages infiltrating the interstitium, and also in and around the glomeruli in the kidney of the normal saline-treated MRL/lpr mice (Fig 4A1). Inversely, in 1.2 and 0.6 mg/10g T-96 groups, the expression of CD68 was obviously down regulated as compared with the normal saline-treated MRL/lpr mice (Fig 4A2, 4A3 and 4C; both p < 0.01). Since IL23, TNF-α, COX-2 and ICAM-1 play essential roles in inflammatory responses, we also examined the levels of these pro-inflammatory mediators in the kidneys. As expected, an enhanced expression of IL23 was clearly detected in the tubules and glomeruli in the normal saline-treated MRL/lpr mice (Fig 4B1 and 4D). In contrast, T-96 from 1.2 to 0.3 mg/10g and Kang lang chuang san substantially reduced expression of IL23 in the tubules and glomeruli in a concentration-dependent manner (Fig 4B2–4B5 and 4D; all p < 0.001). Moreover, a faint expression of IL23 was observed in the tubules and glomeruli of prednisone–treated MRL/lpr mice, with a remarkable decline in the mean density of IL23 (Fig 4B6 and 4D; p < 0.001). Furthermore, the effects of 1.2, 0.6 mg/10g T-96 and prednisone treatment were comparable (Fig 4D, both p > 0.05). As indicated in Fig 5A1, 5B1 and 5C1, TNF-α was localized predominantly in the tubules, whereas COX-2, ICAM-1 were expressed mainly in the glomeruli in normal saline-treated MRL/lpr mice. TNF-α was effectively suppressed by T-96 from 1.2 to 0.3 mg/10g in the tubules of MRL/lpr mice as compared with the normal saline-treated MRL/lpr mice (Fig 5A2–5A4 and 5D, all p < 0.05). Furthermore, treatments of 1.2 and 0.6 mg/10g T-96 significantly reduced COX-2 expression in the glomeruli relative to normal saline-treated MRL/lpr mice (Fig 5B2, 5B3 and 5E; 1.2 mg/10g T-96 p < 0.01 and 0.6 mg/10g T-96 p < 0.05). But 0.3 mg/10g T-96 didn’t significantly reduce TNF-a expression (Fig 5B4 and 5E; p > 0.05). In addition, ICAM-1 was significantly inhibited by 1.2 mg/10g T-96 compared with the normal saline-treated MRL/lpr mice (Fig 5C2 and 5F; p < 0.05). But 0.6 and 0.3 mg/10g T-96 didn’t reduce ICAM-1 expression (Fig 5C3, 5C4 and 5E; both p > 0.05). Our results also indicated that kang lang chuang san markedly reduced the production of TNF-α and ICAM-1 in renal tissues, but it did not reduce COX-2 expression (Fig 5A5, 5B5, 5C5 and 5D and 5E; TNF-α p < 0.01, COX-2 p > 0.05, ICAM-1 p < 0.05). When compared with the normal saline-treated MRL/lpr mice, the secretions of TNF-α, COX-2 and ICAM-1 were significantly reduced in renal tissues of prednisone-treated mice (Fig 5A6, 5B6, 5C6 5D and 5E; all p < 0.05). Collectively, these findings indicate that T-96 is an effective therapy to antagonize renal inflammation during the progression of LN.

### T-96 treatment inhibited the activation of NF-κB signal pathway

**Fig 6:**
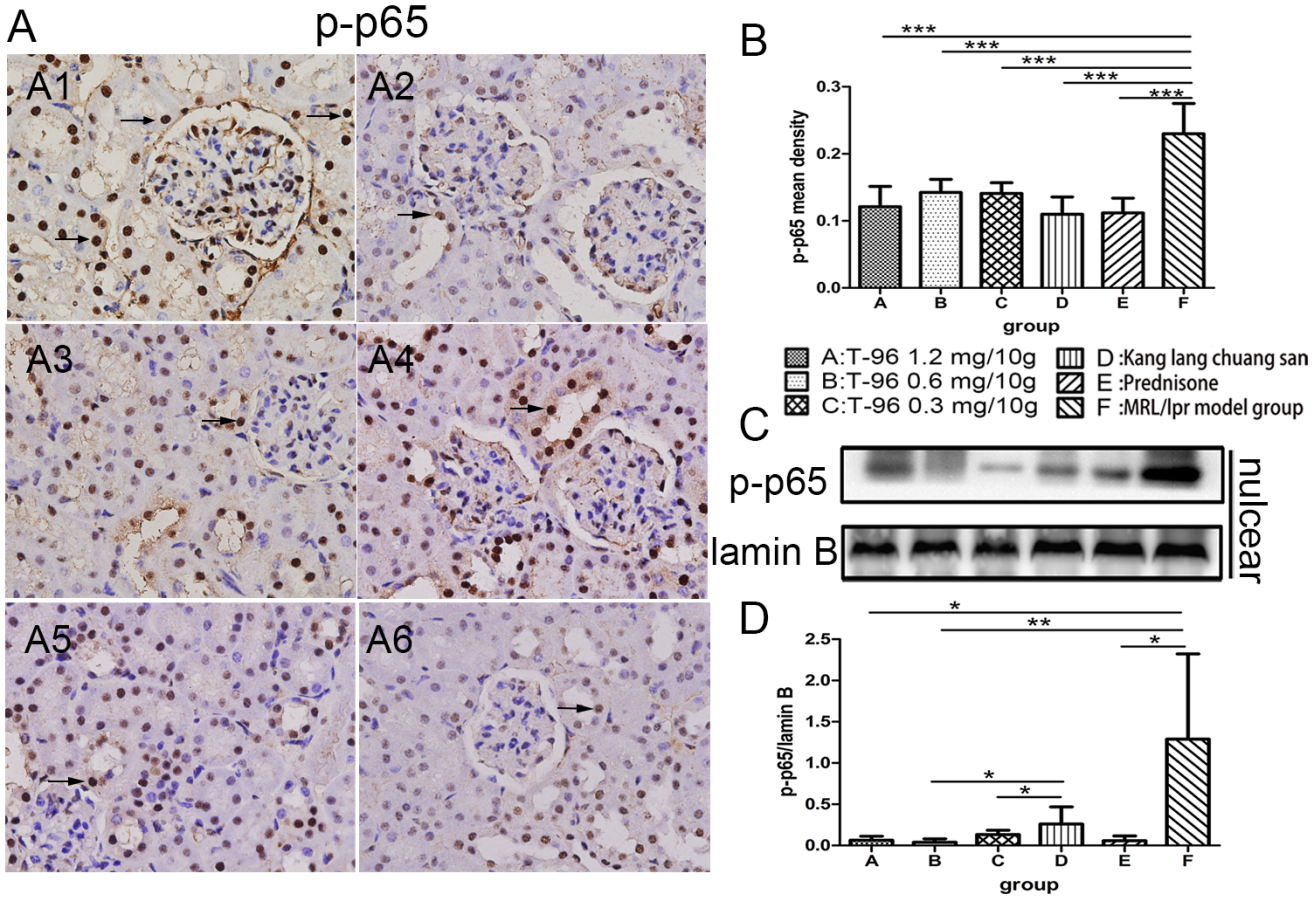
Effect of T-96 on inhibiting phosphorylation of NF-κB p65. (A) NF-κB phosphorylated-p65 (p-p65) was measured on paraffin sections of the kidneys at week 8 by immunohistochemistry (10 x 40). NF-κB p-p65 was indicated by arrows. (B) Mean density of nuclear p-p65 was measured by Image-Pro Plus v 6.0. (C) Further analysis was to measure nuclear p-p65 by Western blot. (D) The Western blot of p-p65 in the nuclear were subjected to semi-quantitative analysis by Image J. Data were expressed as mean ± SD. * indicates P < 0.05, ** indicates P < 0.01, *** indicates P < 0.001. Western blot were repeated > 3 times.

**Fig 7:**
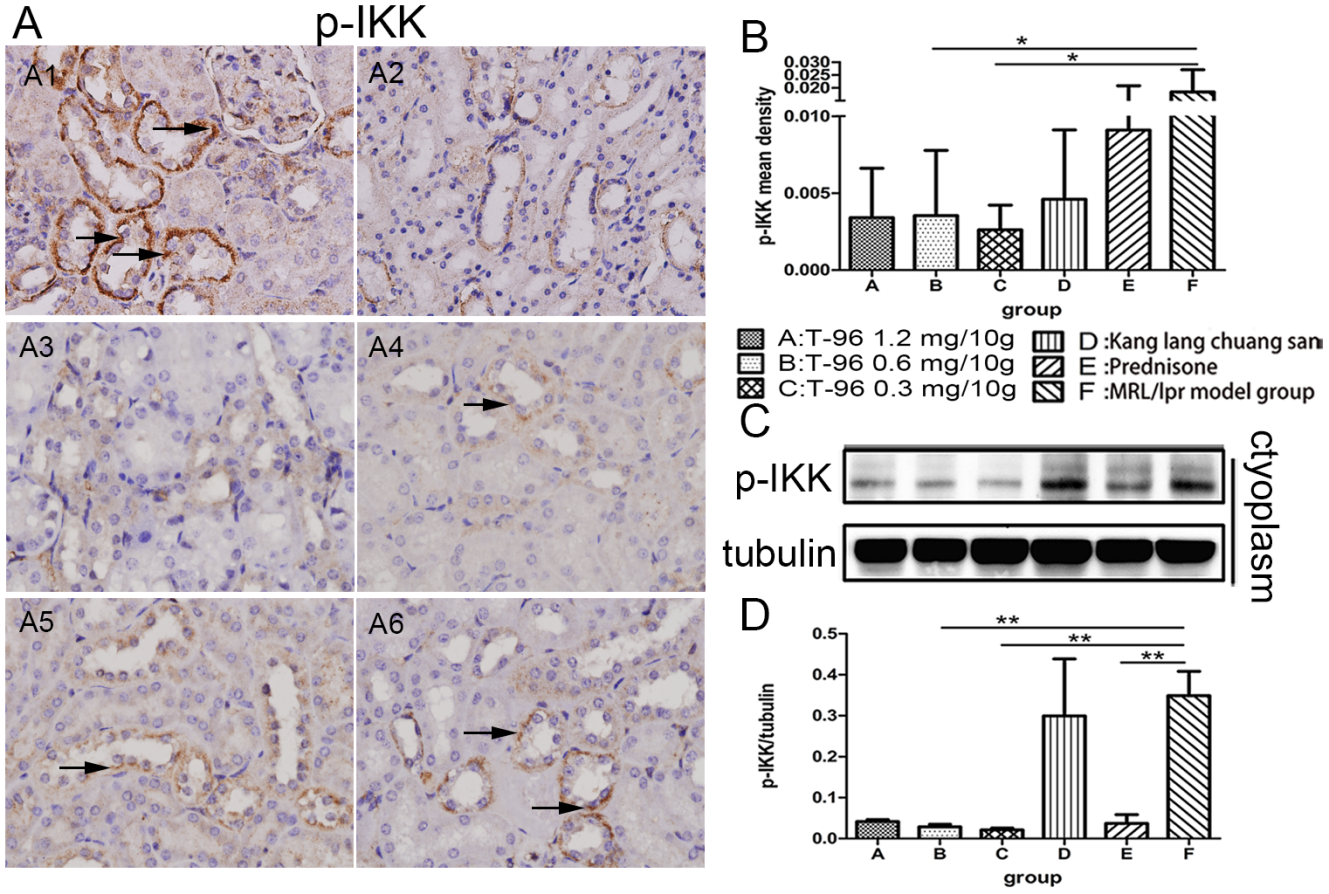
Effect of T-96 on inhibiting phosphorylation of IKK. (A) Cytoplasmic phosphorylated-IκB kinase (p-IKK) was measured on paraffin sections of the kidneys at week 8 by immunohistochemistry (10 x 40). (B) Mean density of cytoplasmic p-IKK was measured by Image-Pro Plus v 6.0. (C) Further analysis was to measure cytoplasmic p-IKK by Western blot. (D) The Western blot of p-IKK in the cytoplasm were subjected to semi-quantitative analysis by Image J. Data were expressed as mean ± SD. * indicates P < 0.05, ** indicates P < 0.01, *** indicates P < 0.001. Western blot were repeated > 3 times.

Because the NF-κB signal pathway plays a key role in orchestrating inflammatory response, we used IHC and Western blotting to measure the effect of T-96 treatments on the activation of NF-κB. Consistent with our IHC results in which pronounced expression of nuclear p-p65 was observed in the tubular epithelial cells and glomerular endothelial and mesangial cells, nuclear p-p65 was readily detected by Western blot in normal saline-treated MRL/lpr mice (Fig 6A1 and 6B–6D). Conversely, nuclear p-p65 expression was evidently decreased in mice treated with 1.2 and 0.6 mg/10g T-96 but not 0.3 mg/10g T-96 (Fig 6A2–6A4 and 6B–6D). IHC revealed that kang lang chuang san significantly restricted phosphorylation of nuclear p65 compared to normal saline-treatment (Fig 6A5 and 6B; p < 0.001), though Western blotting showed no significant inhibition (Fig 6C and 6D). Additionally, prednisone treatment showed an occasional expression of nuclear p-p65 relative to normal saline-treatment (Fig 6A6 and 6B–6D). There was no significant difference between T-96 treatment and prednisone treatment in the levels of nuclear p-p65 as measured by IHC and Western blotting (Fig 6B and 6D). As shown in Fig 7A1 and 7B, a predominant expression of cytoplasmic p-IKK was observed in the tubular epithelial cells of normal saline-treated MRL/lpr mice, a finding that is consistent with the highest ratio of p-IKK to tubulin by Western blot (Fig 7C and 7D). Except for 1.2 mg/10g T-96, rare expression of cytoplasmic p-IKK was identified by IHC in the tubular epithelial cells of mice treated with 0.6 and 0.3 mg/10g T-96 (Fig 7A2–7A4 and 7B), as confirmed by Western blot (Fig 7C and 7D). In contrast, Kang lang chuang san treatment didn’t significantly suppress phosphorylation of cytoplasmic IKK compared to normal saline-treatment (Fig 7A5 and 7B-7D; p > 0.05). Moreover, mice treated with prednisone displayed a decreasing expression of cytoplasmic p-IKK with no significance by IHC (Fig 7A6 and 7B); however, the Western blot results of p-IKK indicated a reduction in contrast to the normal saline-treated MRL/lpr mice (Fig 7C and 7D). These findings demonstrated that NF-κB was activated in renal tissues of MRL/lpr mice, and suggested that T-96 may block NF-κB activation, thus suppressing the expression of target genes in this pathway.

## Discussion

In this study, we explored the anti-inflammatory and immunosuppressive properties of T-96 in mice with LN. Our data showed that T-96 significantly inhibited the activation of NF-kB in the kidneys of MRL/lpr mice. In addition, T-96 reduced the secretion of pro-inflammatory mediators such as TNF-α, COX-2 and ICAM-1. Moreover, T-96 suppressed progression of proteinuria, a biomarker of the kidney inflammation and injury that is the major cause of mortality in the MRL/Lpr model of SLE. Macrophage infiltration and IL23 expression were also secondary to NF-κB activation, providing an additional therapeutic mechanism for T-96.

NF-κB activation has been previously reported in renal tissue, including glomerular epithelial, mesangial cells, renal tubular as well as interstitial cells in human LN, along with over-production of downstream pro-inflammatory mediators and upstream signaling molecules, which has demonstrated to play a pathological role in LN [30,31]. Our study showed that nuclear p-p65, the main active component of NF-κB, was significantly reduced by the 1.2 and 0.6 mg/10g T-96 treatment. Moreover, the upstream component p-IKK was also remarkably decreased by 0.6 and 0.3 mg/10g T-96. Thus, NF-κB might represent a target for T-96 and provide a novel treatment strategy.

In our study, we found that NF-κB-mediated secretions of pro-inflammatory mediators such as TNF-α, ICAM-1 and COX2 were remarkably inhibited by T-96 treatment. TNF-α is the cardinal pro-inflammatory cytokine primarily produced by macrophages upon injury, which then stimulates the activation of the classical NF-κB pathway and in turn regulates the production of more TNF-α [16,32]. Thus, T-96 represents a therapeutic agent that blocks proximal cytokines such as TNF-α, thereby limiting NF-κB activation and inhibit the inflammatory cascade seen in LN. Elevated bioactive TNF-α is expressed in the renal tissues and also found in the circulation during active LN, and is likely involved in the pathogenesis of LN. In terms of its precise role in the development of LN, it’s reported that TNF-α preceded the inflammatory response to glomerular immune complex deposition, by means of attracting macrophages infiltration to periglomeruli and interstitium via up-regulating a set of macrophage-related chemokines [33]. What’s more, anti-dsDNA antibody-mediated induction of TNF-α increased IL-1β and IL-6 secretions in mesangial and proximal renal tubular epithelial cells, and provided an impetus for apoptosis in resident renal cells [34]. Emerging evidence suggests that increased expression of ICAM-1 was found in the glomerular mesangium and on the endothelium in patients and mice with active LN, and reflected the severity of LN [35]. Of note, COX-2 was reported to over-express in lymphoid cells of lupus mice such as autoimmune T cells, B cells and macrophages, thus activating their functions such as promoting presentation of the major lupus autoantigen and accelerating autoantibody production. Some drugs, such as apigenin and celecoxib aiming at COX-2 and NF-κB in activated autoimmune cells, are reported to have beneficial effects in LN [36,37]. These observations further support our proposal that T-96 may alleviate inflammation response in LN by restricting the activation of NF-κB and reducing the secretion ofits downstream pro-inflammatory mediators.

It is believed that the infiltration of macrophages in the interstitium and glomeruli is a prominent feature in both human and mice LN, and it is also regarded as the most useful marker of SLE and renal activity. Drawn the sites of inflammation, macrophages are activated by systemic CSF-1 (Colony-stimulating factor-1) toward “inflammatory” populations, promoting a more rapid accumulation of intrarenal macrophages [38]. These accumulating macrophages secrete inflammatory cytokines and chemokines, including IL23, TNF-α and COX-2 that contribute to the apoptosis of tubular epithelial cells and drive glomerulonephritis. In addition, regarded as important antigen-presenting cells, macrophages play a vital role in the presentation of nucleosome-derived autoantigens to autoreactive T cells in mice with lupus [39], and hyperactive APCs are a characteristic feature of lupus [40,41]. Consistent with these researches, we found that CD68^+^ macrophage predominantly infiltrated in the interstitium, and also in and around the glomeruli in the kidney of the MRL/lpr mice. Moreover, NF-κB, has been shown to drive macrophages toward a specific phenotype in a multitude of inflammatory diseases through interacting with other transcription factors [42]. Still other studies have indicated that NF-κB acts in a central role between macrophages and renal cells by connecting pro-inflammatory mediators [43–45]. In this context, our observations demonstrated that 1.2 and 0.6 mg/10g T-96 treatment effectively suppressed the infiltration of macrophages and the production of macrophages-related mediators, IL23 and COX-2. This was supported by the inhibition of NF-κB in the kidney of MRL/lpr mice following the T-96 treatment. However, which type of macrophage was affected by T-96 and the definite mechanism involved need to be further explored.

It is generally accepted that the levels of anti-dsDNA antibody in serum remain widely utilized both to help establish the diagnosis of SLE and to predict nephritis activity [46]. There are obviously sufficient reasons for believing that these antibodies may in some instances be involved in the pathogenesis of LN [47–49]. Our study showed that 1.2 and 0.6 mg/10g T-96 treatment remarkably inhibited the levels of anti-dsDNA antibody in serum to levels comparable to prednisone treatment.

In conclusion, T-96 treatment ameliorates the progression of proteinuria and renal pathology in LN. These effects accompany by inhibiting activation of NF-κB, suppressing release of pro-inflammatory mediators, and by preventing macrophage infiltration in the kidney of experimental LN. As such, T-96 represents promising therapy aimed at preventing the progression of LN.
